# Development of a Mobile App (MyLepto App) to Improve Knowledge, Attitude, and Practice Regarding Leptospirosis Among Wet Market Workers in Selangor, Malaysia: Protocol for a Quasi-Experimental Study

**DOI:** 10.2196/75809

**Published:** 2026-01-23

**Authors:** Mas Norehan Merican Aljunid Merican, Zaleha Md Isa, Rozita Hod, Roszita Ibrahim, Zamtira Seman, Rusdi Abd Rashid

**Affiliations:** 1 Department of Public Health Medicine Faculty of Medicine Universiti Kebangsaan Malaysia Jalan Yaacob Latif, Bandar Tun Razak, Cheras, Kuala Lumpur Malaysia; 2 Sector for Biostatistics & Data Repository National Institutes of Health Ministry of Health Malaysia Shah Alam Malaysia; 3 Department of Psychological Medicine Faculty of Medicine University Malaya Kuala Lumpur Malaysia

**Keywords:** leptospirosis, mobile health app, Health Belief Model, wet market workers, public health intervention

## Abstract

**Background:**

Leptospirosis is the most common zoonotic cause of mortality, with most of its burden occurring in tropical regions and low-income countries. It is endemic in Southeast and South Asian nations. Leptospirosis outbreaks occur after natural disasters. In Malaysia, the e-notification system of the Communicable Diseases Control Information System recorded 5217 leptospirosis cases in 2019 with 32 fatalities. The incidence rate was 15.61 per 100,000 people. Male individuals comprised 67% of leptospirosis cases, while people aged 25 to 55 years accounted for 45% of the cases. Information and perception are crucial in influencing positive behavior. Nonetheless, information on urban and rural people’s knowledge, attitude, and practice (KAP) regarding the incidence of leptospirosis is limited.

**Objective:**

We aimed to develop a mobile app with information on leptospirosis and measure its effectiveness in improving KAP regarding leptospirosis among wet market workers in Selangor, Malaysia.

**Methods:**

A 3-phase study will be conducted and includes development of a mobile app containing information about leptospirosis, analysis of its acceptability, and application of the intervention. Participants will be recruited based on specific inclusion criteria by using purposive sampling. Four wet markets in Hulu Langat district, Selangor, will be selected according to a list provided by local municipal councils. The respondents from each selected wet market will be workers aged 18 years and older. Mobile app development will begin with an idea description, storyboard creation, and content approval through the nominal group technique. The mobile app content will be constructed using the Health Belief Model theory. Subsequently, the usability of the mobile app prototype will be evaluated using the validated Malay version of the System Usability Scale questionnaire for the evaluation of mobile apps. This protocol entails a 12-week intervention stage, in which the baseline assessment is regarded as a pretest evaluation and the follow-up assessment as a posttest evaluation. Participant selection will be based on the inclusion and exclusion criteria. This study will incorporate a set of validated questionnaires created by a group of leptospirosis experts. The validated questionnaire will comprise 9 sections with open-ended questions on sociodemographic data, KAP, and mobile app requirements.

**Results:**

Mobile app development and usability testing were completed between January 2024 and March 2025. Participant recruitment is scheduled in April to May 2025 after submission of this manuscript, with the 12-week intervention and data collection running from May to July 2025. As of manuscript submission, recruitment, data collection, and data analysis have not yet begun. Data analysis is expected to be completed by September 2025, and results are anticipated for publication in late 2025.

**Conclusions:**

Due to the high number of reported leptospirosis cases in the Hulu Langat district, Selangor, this intervention study will be conducted there. The development of the mobile app may contribute to improving wet market workers’ KAP regarding leptospirosis.

**International Registered Report Identifier (IRRID):**

PRR1-10.2196/75809

## Introduction

### Background

Leptospirosis is a neglected tropical illness with a large global health burden and is the most common cause of zoonotic disease and mortality. The main leptospirosis disease burden occurs in tropical regions and the poorest countries. Southeast Asia recorded the second-highest leptospirosis incidence after Oceania, with an estimated 1.03 million cases and 58,900 fatalities yearly. Given the dearth of current information, these statistics may have been underestimated. Leptospirosis is endemic in South and Southeast Asia (regions with humid subtropical and tropical climates), with outbreaks occurring after natural disasters [1], especially after a heavy rainy season or flooding.

**Figure 1:**
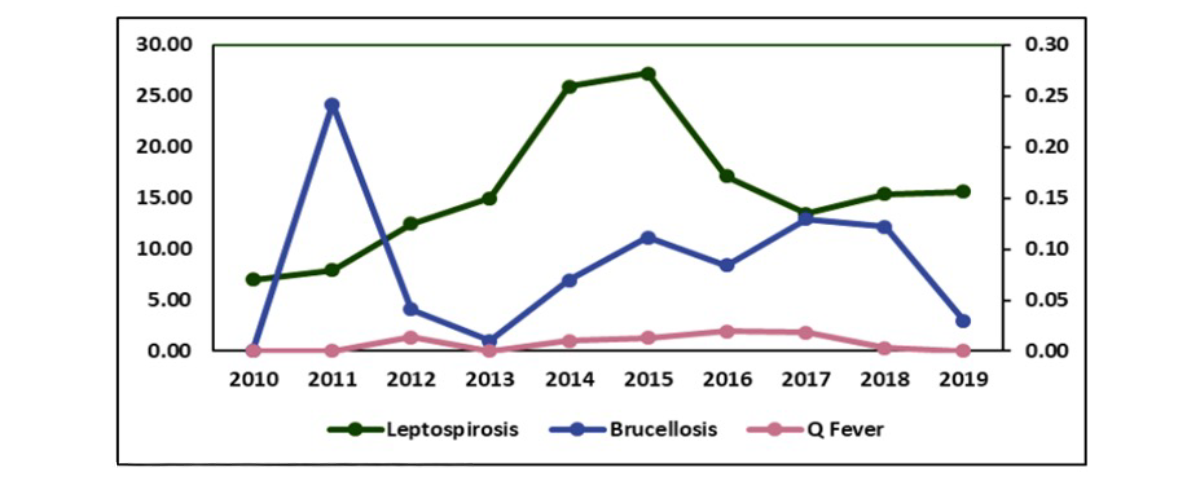
Incidence rate for brucellosis, Q fever, and leptospirosis per 100,000 population in Malaysia from 2010 to 2019 (reproduced from Ministry of Health Malaysia [2], with permission from Ministry of Health Malaysia).

Malaysia is close to the equator and experiences significant rainfall, especially during the monsoon season. Hence, Malaysia is prone to being leptospirosis-endemic. Local data indicated that Malaysian leptospirosis cases increased from 1.03 cases per 100,000 people in 2004 to 30.2 cases per 100,000 people in 2015. Furthermore, the e-notification system of the Communicable Diseases Control Information System recorded 5217 leptospirosis cases in 2019, with 32 fatalities (case fatality rate: 0.6%), while the incidence rate was 15.61 per 100,000 people (Figure 1) [2].

### Current Leptospirosis Situation in Malaysia

**Figure 2:**
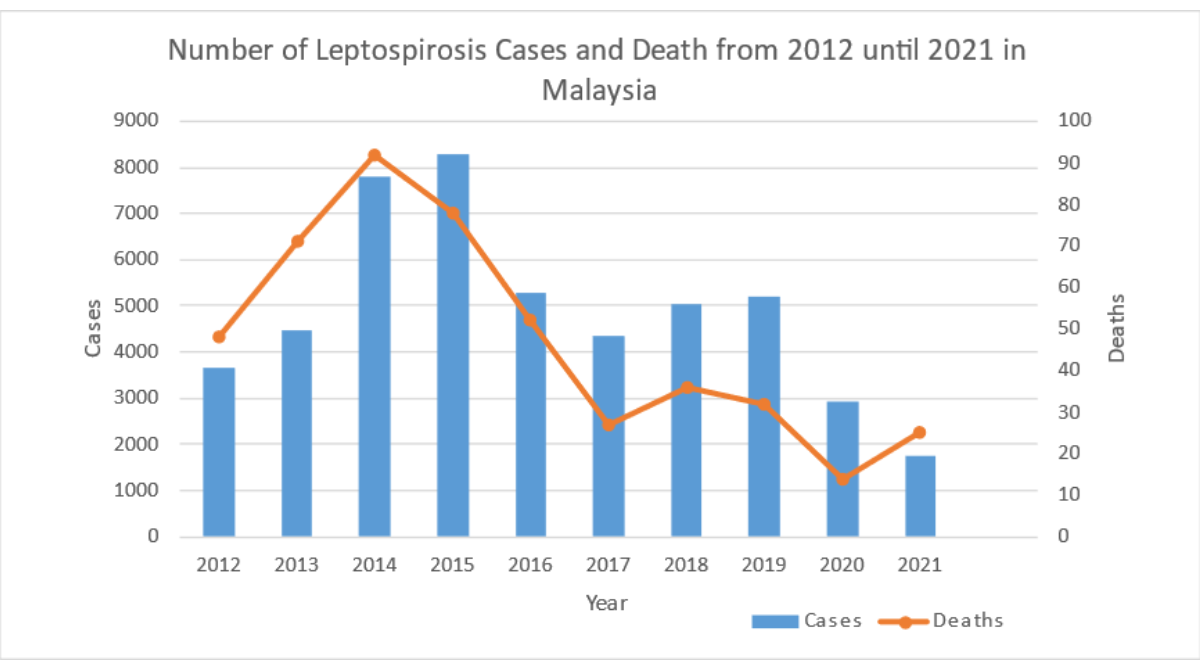
Number of leptospirosis cases and deaths in Malaysia from 2012 to 2021 (reproduced from Ministry of Health Malaysia [2], with permission from Ministry of Health Malaysia).

In Malaysia, leptospirosis must be reported under the Prevention and Control of Infectious Diseases Act of 1988 as of December 9, 2010 [3]. The incidence of leptospirosis in Malaysia has markedly increased for more than a decade, resulting in a significant number of fatalities. In 2020, 2914 leptospirosis cases (incidence rate: 8.63 per 100,000 population) and 38 deaths were recorded (death rate: 0.1 per 100,000 population). Eight leptospirosis outbreaks were recorded in 2020, of which 50% involved households [3]. A total of 1763 leptospirosis cases (incidence rate: 4.81 per 100,000 population) and 25 deaths were recorded in 2021 (death rate: 0.07 per 100,000 population; Figure 2) [4-13]. The occurrence of some zoonoses, particularly leptospirosis, declined during the COVID-19 pandemic, which might have been linked to the Movement Control Order, school closures, and reduced recreational activities. Nevertheless, there is a great need for research and development to identify leptospirosis mitigation methods and address the increasing number of fatalities.

### Risk Groups

Exposure to *Leptospira* in contaminated environments, such as wet markets, increases the risk of leptospirosis. An effective antibody against the disease is required to mitigate the leptospirosis infection rate [14]. Wet market workers were suggested to be at risk of contracting leptospirosis, as proven by the high seroprevalence of leptospirosis [15]. In addition, high-risk planters recorded a high seroprevalence of leptospiral antibodies (28.6%) [16], whereas the seroprevalence among wet market workers was 20% to 33.6% [15]. Thus, recognizing interventions to improve leptospirosis knowledge, attitude, and practice (KAP) would aid wet market workers in effectively addressing their risks.

### Mobile App Use in Malaysia

**Figure 3:**
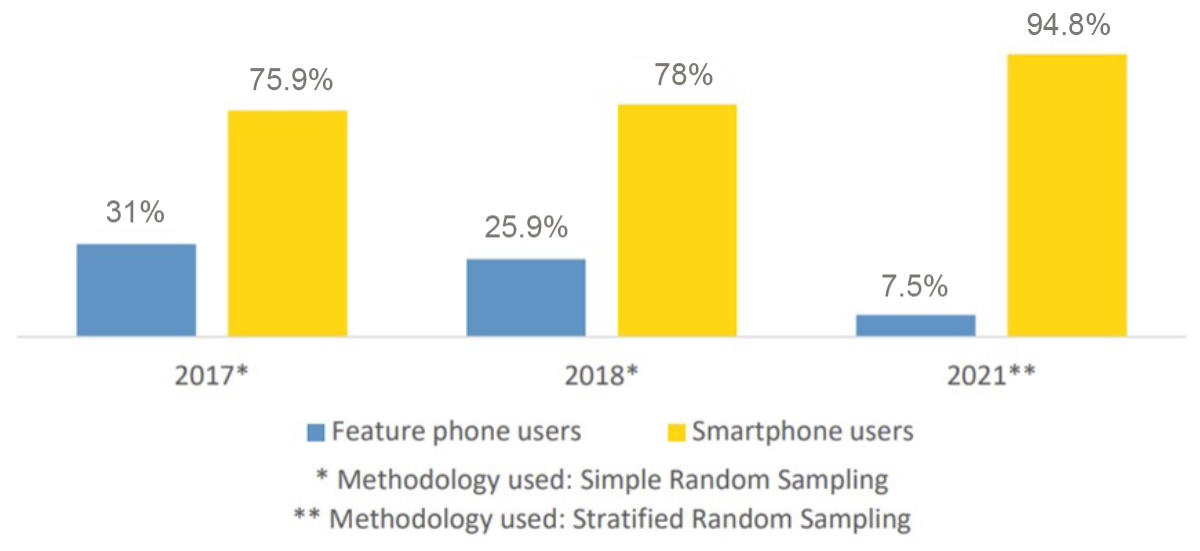
Percentage distribution of smartphone and feature phone users in Malaysia from 2017 to 2021 (reproduced from Malaysian Communications and Multimedia Commission [17], with permission from Malaysian Communications and Multimedia Commission).

The Malaysian Communications and Multimedia Commission Hand Phone Users Survey 2022 reported 94.8% smartphone penetration in Malaysia in 2021 [17] (Figure 3). However, the percentage of people using feature phones is decreasing, with only 7.5% expected to do so by 2021.

**Figure 4:**
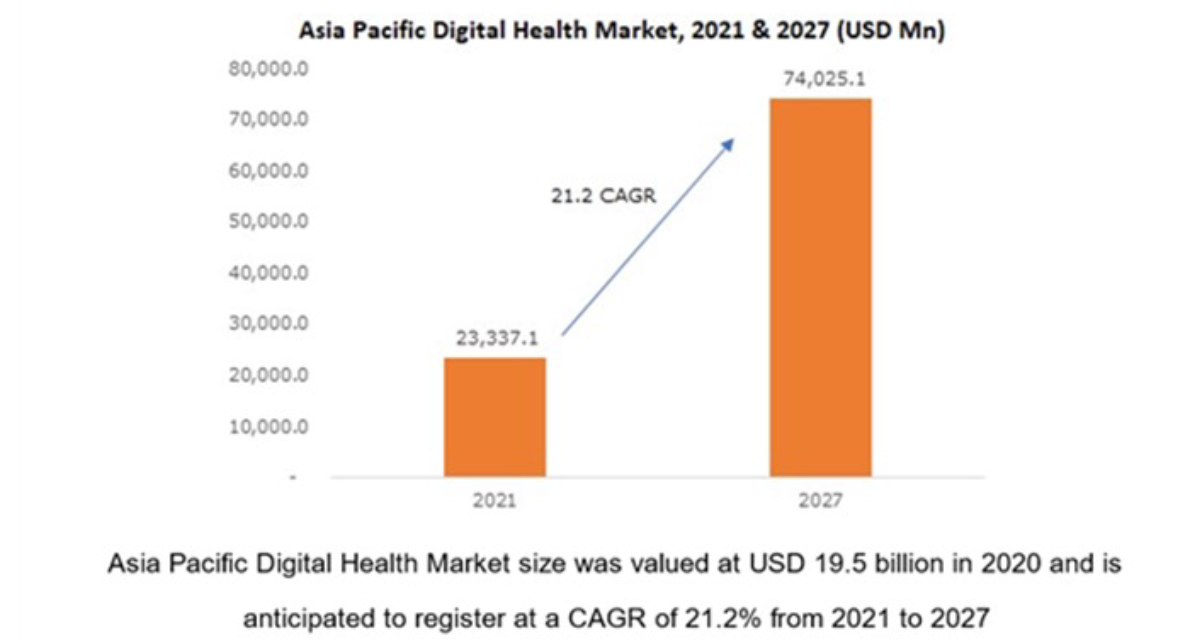
Projected growth of the Asia Pacific digital health market, 2021–2027. Source: Data adapted from Graphical Research [18].

Practicality and commercialization are prevalent in digital health care and awareness, especially after the COVID-19 pandemic outbreak. The 2027 projection indicates that the Asia-Pacific digital health care market value is expected to increase by 21.2%, which would exceed RM 50 billion (US $1=RM 4.08) (Figure 4) [18].

These data reflect the need for research to improve leptospirosis KAP and prevent it among wet market workers in the Hulu Langat district, Selangor. A mobile app is expected to be beneficial for raising leptospirosis awareness among Malaysians, as it will enlighten the community about the disease and its prevention [19].

### Why Is This Study Important?

While the availability of scientific data has improved the understanding of leptospirosis in Malaysia, most cases in Selangor, Malaysia, have not been reported. This condition has resulted from the metropolitan community’s lack of awareness of the disease [20]. Knowledge and perception are significant motivating factors of constructive behavior [21]. Unfortunately, research on leptospirosis KAP in Malaysia is subject to the lack of understanding of leptospirosis prevalence among urban and rural populations [22]. Thus, this protocol describes a study that aims to develop a mobile app to educate the community on leptospirosis and its prevention, and substitute current information dissemination methods [19].

### Objectives

Workers in the agriculture and domestic animal industries are at high risk of contracting leptospirosis. Furthermore, rodent presence and environmental hygiene in wet markets have been related to leptospirosis. Hence, the primary study aim is to identify the requirements for developing a mobile app as a leptospirosis prevention intervention among wet market workers in Hulu Langat. The secondary study aim is to design and develop a mobile app with leptospirosis information to enhance KAP among wet market workers in Hulu Langat and to measure the improvements in the workers’ KAP regarding leptospirosis after a 12-week intervention through pretest and posttest evaluations.

## Methods

### Study Design and Study Area

The study will comprise 3 phases. The first phase will develop a mobile app, the MyLepto app. The second phase will analyze the acceptability of the mobile app, while the third phase will involve the intervention with a follow-up of 12 weeks. The study will be conducted in Hulu Langat and involve preintervention and postintervention evaluations. This study will use a quasi-experimental design. Eligible respondents from 4 wet markets in Hulu Langat will be selected through simple random sampling [23].

This study will be conducted in Hulu Langat, which is well known for having high incidence rates of leptospirosis due to the large concentration of wet markets and other high-risk locations. Hulu Langat was selected given its numerous wet markets where workers regularly encounter possible leptospirosis sources, such as tainted water and rodent populations [24].

### Target Population

The target population will be wet market workers in Hulu Langat, who are frequently exposed to risk factors for leptospirosis due to their jobs. High population densities, frequent contact with organic waste, and close contact with live animals are all characteristics of wet markets that increase the risk of zoonotic illnesses, such as leptospirosis [25].

### Sample Population

The sample population will comprise wet market workers from 4 selected locations in Hulu Langat who fulfill the inclusion criteria.

### Inclusion Criteria

This study will include Malaysian wet market workers aged 18 years or above who have worked at 1 of the 4 selected Hulu Langat wet markets for at least 6 months. The potential participants must also have access to a mobile device compatible with the MyLepto app and be willing to interact with it to participate in the study. All potential participants will be required to provide informed consent before their inclusion in the study.

### Exclusion Criteria

The exclusion criteria are people with cognitive or physical disabilities that would make it difficult for them to use a mobile app or complete the study. People who have participated in comparable leptospirosis-related intervention programs within the last 6 months will be excluded to reduce bias. Furthermore, people who do not frequent the 4 wet markets, such as temporary or part-time workers who work less than 2 days per week, will also be excluded. Wet market workers will also be excluded from the study if they refuse to participate or withdraw their consent at any time.

### Sample Size Calculation and Sampling Technique

An effect size of 0.26 was derived based on the means (SDs) before and after the intervention: 75.2 (13.29) and 78.65 (13.19), respectively [15]. A calculated sample size of 118 was obtained using 2 dependent means (matched pairs) at a 5% level of significance and 80% power. With the assumption of a 10% dropout rate, the final sample size needed for the study is 130. The figure was obtained using G*Power (version 3.1.9.4).

Participants will be selected using stratified random sampling. Four wet markets in Hulu Langat will be purposively selected based on high-risk profiles and logistical feasibility. Within each market, workers will be stratified according to job roles (vendors, cleaners, and handlers). A proportional random sample will be drawn from each stratum to reduce selection bias and enhance representativeness. Local councils will assist in verifying employment records.

### Intervention and Timeline

Textbox 1 [26,27] describes how the 12-week intervention will be conducted.

**Activities and assessments.**

**Timeline**

- Wk 0 (baseline): administration of pretest knowledge, attitude, and practice questionnaire [26] and evaluation of baseline System Usability Scale (SUS) [27]
- Wk 1-12 (intervention): weekly issuance of app-based educational modules, quizzes, and reminder announcements
- Wk 6 (midpoint): interim SUS evaluation of usability
- Wk 12 (postintervention evaluation): administration of the posttest knowledge, attitude, and practice questionnaire, final evaluation of SUS, and the field observation checklist for preventive practices

### Phase 1: Mobile App Development

**Figure 5:**
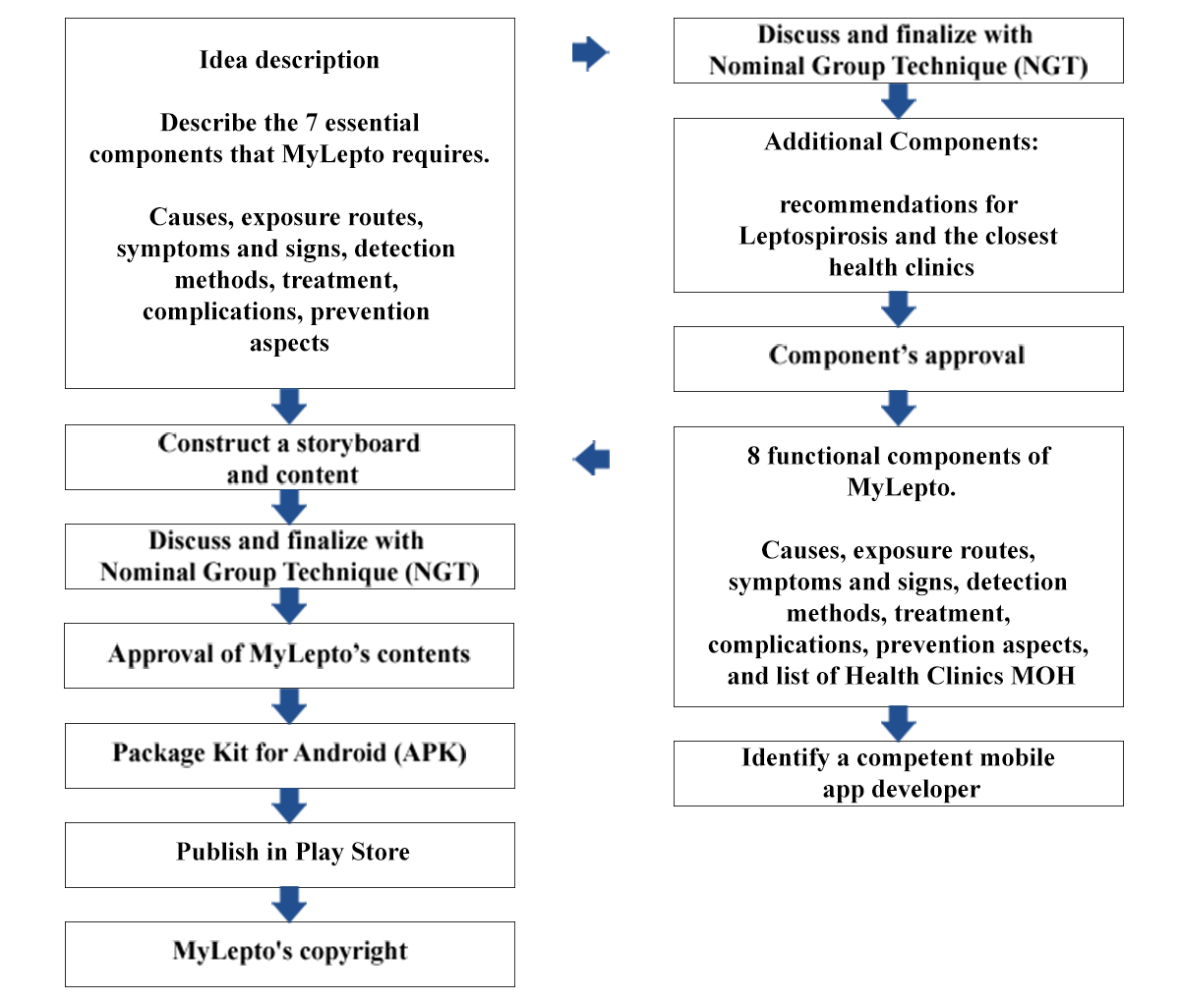
Development process of the MyLepto mobile application. MOH: Ministry of Health.

The mobile app development involves 7 main steps. Step 1 involves the idea description, followed by storyboard creation (step 2). In step 3, the content is to be approved by the nominal group technique (NGT), followed by finalizing the content in the Android package file format. The last 2 steps are publication in the Google Play Store and copyright acquisition from the Malaysian Intellectual Property Corporation. Figure 5 depicts the general design process of the mobile app.

The NGT will be used in the development of the mobile app content. Wet market workers will act as the target users to identify vital communication strategies and health information, including the elements for a user-friendly mobile app. In addition, public health specialists, family medicine specialists, and health inspectors will also be included as the target users.

The NGT is a structured meeting that aims to create a systematic process for collecting qualitative data from the target groups most intimately associated with the current issue [28]. In 2006, the Centers for Disease Control and Prevention stated that a small group discussion would aid in decision-making. The participants will be instructed to answer questions presented by a moderator or a researcher to obtain data for the NGT. Next, the participants will be instructed to rank the recommendations or thoughts arising from each group member [29]. This method was created by Delbecq and Van de Ven and comprises 4 important phases: silent generation, round robin, clarification, and voting (rating or ranking) [30].

### Content Development

The mobile app content will be developed using the Health Belief Model (HBM) theory [31]. The mobile app will undergo several content development stages (Figure 5). The first step reviews all available studies on leptospirosis, including the signs, symptoms, transmission methods, related complications, prevention, and control measures. This step will also gather information on the mobile app features for health education and intervention. The next step in identifying the relevant content for the target users will be based on the appropriate information synthesized. For example, users may find it difficult to express their thoughts if they are unfamiliar with the mobile app features. Hence, researchers will determine the features that users anticipate in a mobile app based on existing data.

The mobile app incorporates HBM constructs to encourage preventive behavior. Interactive modules and animated visuals on leptospirosis risks and complications will address perceived susceptibility and severity. Practical prevention tips will highlight the benefits of simple hygiene practices, while an interactive frequently asked questions section will address common barriers. Cues to action will be provided via weekly notifications and outbreak alerts. Finally, self-efficacy will be supported through step-by-step guidance and real-life examples.

### Phase 2: Mobile App Acceptability Analysis

The usability of the mobile app prototype will be evaluated in Malay (Skala Kebolehgunaan Aplikasi Mudah Alih) using the validated Malay version of the System Usability Scale (SUS) questionnaire for the evaluation of mobile apps. The SUS questionnaire was originally designed in English and comprises 10 items, and will be translated into Malay using forward-backward translation. Two translators (1 linguistic expert and 1 field expert) will translate the questionnaire from English to Malay, while another 2 experts (1 linguistic expert and 1 field expert) will back-translate the questionnaire from Malay to English. Finally, the researchers and translators will discuss to finalize the Malay version of the questionnaire. The Malay version will be validated to test the mobile app usability, as described by Mohamad Marzuki et al [26]. The response score will be determined using a 5-point Likert scale, ranging from 1 (strongly disagree) to 5 (strongly agree). The total score (range 0-100) will be determined by the sum of all item scores and multiplied by 25. The original author suggested a standard usability score of 68 to signify the good usability of an app [32].

The data used in constructing the mobile app were obtained from trusted sources. Initially, information on the introduction, prevention, causes, and treatment of leptospirosis was collected from the Ministry of Health Malaysia MyHealth Portal [33]. Subsequently, the information was reviewed by epidemiology experts, comprising public health and family medicine specialists.

### Phase 3: Intervention Study

**Figure 6:**
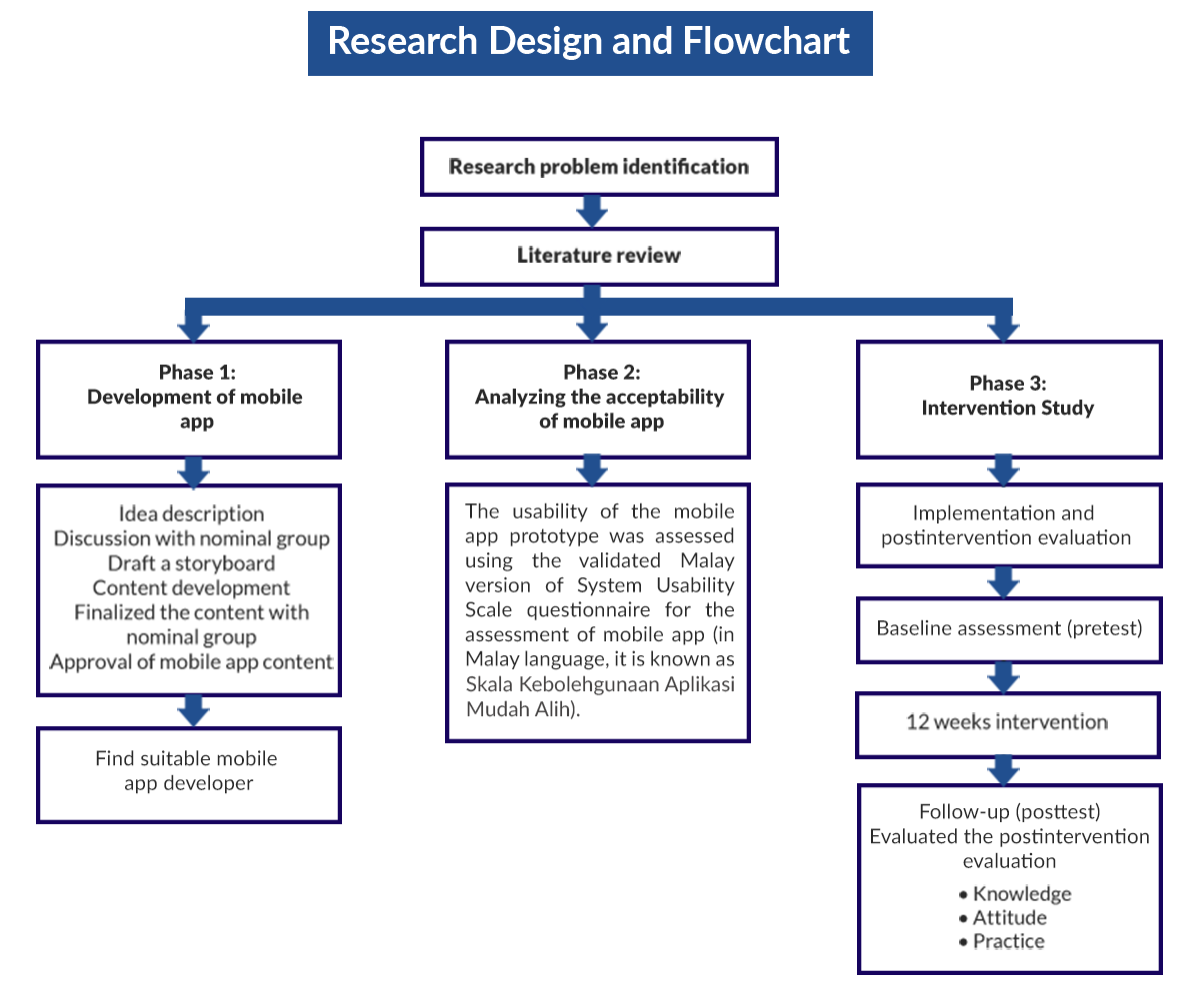
Methods of data collection during the intervention study.

The study will be conducted in Hulu Langat and involve preintervention and postintervention evaluations. Figure 6 demonstrates that the main procedures in the 12-week intervention stage and study protocol involve a pretest evaluation characterized by baseline assessment, followed by a posttest evaluation. Participants will be recruited based on specific inclusion and exclusion criteria. The local municipal council of the workers’ areas will cooperate with the research team to select and educate the participants on the research objectives during the initial recruitment stage. The researcher will organize a meeting at this stage. Participant engagement will be maintained throughout the 12-week intervention through strategies such as weekly push notifications to prompt the completion of educational modules and quizzes; the use of gamified elements (interactive quizzes with instant feedback); and group engagement through WhatsApp groups organized by the market, where weekly summaries and interactive polls will be shared to sustain motivation and peer interaction.

The evaluation framework includes SUS assessments at weeks 0, 6, and 12 to monitor usability, a workplace behavior checklist at week 12 to observe preventive practices, and process measures (app use data, retention rates, and postintervention interviews) to evaluate implementation effectiveness and identify areas for improvement.

### Questionnaire

This study will use a validated KAP questionnaire on leptospirosis in Malaysia, which will be adopted and modified as needed [27]. The content validity of the study instrument will be ensured by cross-checking and authentication by experts in the field. Before the study begins, the questionnaire will be pretested with 30 wet market workers from Hulu Langat. The questionnaire contains 9 sections with open-ended questions on sociodemographic data and KAP [27,34].

Four of the 9 sections will focus on KAP, including sociodemographic characteristics and place of residence. The participants will be required to disclose their age, gender, ethnicity, household income, and highest level of education.

### Data Collection

The data will be collected through self-administered questionnaires. Typically, the questionnaire will be completed in 30 minutes. This study will be conducted in Hulu Langat and involve preintervention and postintervention evaluations. Measurements will be recorded from May 2025 to July 2025. App analytics will be used to monitor intervention adherence.

The recruitment and screening will be based on specific inclusion and exclusion criteria. The duration of the intervention phase will be 12 weeks, and the follow-up phase will be a posttest phase (Figure 6). The researcher will conduct a meeting with the participants’ leader. During this phase, the leader will cooperate with the research team to select and educate the participants on the research objectives during the initial recruitment stage. The participants will receive a flyer describing the study details and the respondents’ eligibility criteria. Subsequently, the participants will be instructed to complete a validated, self-administered preintervention questionnaire.

### Data Analysis

All data collected will be entered and analyzed using SPSS (version 24.0; IBM Corp). Means and SDs will be presented for numerical variables, while frequencies and percentages will be presented for categorical data. The adjustment in the outcome measurement between the initial and intervention stages will be compared using a paired 2-tailed *t* test to assess the effectiveness of the mobile app. Usability across time points will be assessed using repeated measures ANOVA. The relationships between sociodemographic variables and KAP improvements will be examined using logistic regression.

### Participants and Public Involvement

Wet market workers and an expert group were actively involved in the development process from the beginning to the final stages to ensure that the intervention was applicable, practical, and grounded in behavioral change theories, particularly the HBM. A cross-sectional study of wet market workers was conducted to identify the major gaps in their KAP regarding leptospirosis. Their responses influenced the development of the content of the mobile app. A consensus on the elements and features of the mobile app emerged after an expert group engaged in an NGT session. This process ensured that the mobile app design incorporated end-user requirements and expert-driven insights. During the development stage, wet market workers participated in usability testing sessions to evaluate and enhance the mobile app. During the intervention phase, the wet market workers will assess how effectively the mobile app improves their KAP regarding leptospirosis.

### Ethical Considerations

This study was conducted in accordance with the principles of the Declaration of Helsinki and received ethics approval from the Universiti Kebangsaan Malaysia Research Ethics Committee (UKM PPI/111/8/JEP-2023-069). As the study did not involve data, patients, or facilities under the jurisdiction of the Ministry of Health, approval from the Ministry of Health Medical Research and Ethics Committee was not required. However, the study is registered with the National Medical Research Register (NMRR-ID-23-01054-DVX). Before participation, written informed consent will be obtained from the participants. The data will undergo anonymization and be stored securely. The participants will receive a small token voucher for participation. This study will not use identifiable participant images.

## Results

The development of the mobile app, which incorporates HBM-based content and was validated through the NGT, was completed in January 2024. The study preparation timeline included refinement of the app content and navigation based on usability testing from February to March 2025, followed by a 3-day training workshop for research facilitators, and stakeholder meetings in April 2025. Participant recruitment will be conducted from April to May 2025 through flyers, market briefings, and eligibility screening. The 12-week intervention, which includes baseline and posttest KAP assessments, is scheduled from May to July 2025. Data analysis is expected to be completed by September 2025, and the study results are anticipated to be published by the end of 2025.

## Discussion

### Anticipated Findings

This study aims to develop and evaluate the MyLepto app, which is a mobile health app aimed at improving the leptospirosis-related KAP among Selangor wet market workers. This intervention was guided by the HBM and is expected to increase leptospirosis awareness, improve preventive actions, and reduce occupational risk. The assessment of usability and effectiveness will yield evidence on the role of mobile health interventions in dealing with zoonotic diseases in populations at high risk.

### Comparison With Prior Work

Previous studies have demonstrated the potential of mobile apps in improving health outcomes across chronic disease management and infectious disease prevention. To the best of our knowledge, no mobile intervention has specifically targeted the prevention of leptospirosis among wet market workers to date. Thus, this study bridges an important gap by providing context-specific health education to a vulnerable occupational group. Furthermore, integrating the HBM will align this protocol with established approaches in behavioral science and support its potential to lead to significant change.

### Strengths and Limitations

The strength of this study is its focus on a high-risk population (wet market workers), which ensures that the intervention is relevant and contextually specific. Furthermore, the 3-phase development process enhances the scientific rigor of the app design. Moreover, the inclusion of the validated KAP and SUS questionnaires will enhance the reliability of outcome measurements.

Nevertheless, this study has several limitations. First, the intervention duration (12 weeks) may not capture long-term behavioral alterations, which would require additional follow-up studies. Second, the participants’ differing digital literacy and familiarity with mobile technology may influence their app engagement and, consequently, app effectiveness. Finally, the quasi-experimental design lacks a control group, which may limit causal inferences regarding the effects of the intervention.

### Future Directions and Dissemination

Future research should involve a longer-term follow-up to evaluate continual behavioral alteration and examine the integration of gamification or social features to improve engagement. Furthermore, the public health influence of the intervention may be broadened by expanding it to other high-risk occupational groups, such as agricultural workers or sanitation staff. The plan for dissemination includes sharing the results with local health authorities, publishing the results in peer-reviewed journals, and presenting the outcomes at national and international conferences. If the MyLepto app is effective, it may act as a scalable model for mobile health interventions that target neglected tropical diseases in Malaysia and other countries.

## Abbreviations

HBM: Health Belief Model
KAP: knowledge, attitude, and practice
NGT: nominal group technique
SUS: System Usability Scale

## References

[1] Chadsuthi S, Chalvet-Monfray K, Geawduanglek S, Wongnak P, Cappelle J (2022). Spatial-temporal patterns and risk factors for human leptospirosis in Thailand, 2012-2018. Sci Rep.

[2] (2019). Annual report of the Ministry of Health. Ministry of Health Malaysia.

[3] (2011). Guidelines for the diagnosis, management, prevention and control of leptospirosis in Malaysia. Ministry of Health Malaysia.

[4] (2012). Annual report 2012. Ministry of Health Malaysia.

[5] (2013). Annual report 2013. Ministry of Health Malaysia.

[6] (2014). Annual report 2014. Ministry of Health Malaysia.

[7] (2015). Annual report 2015. Ministry of Health Malaysia.

[8] (2016). Annual report 2016. Ministry of Health Malaysia.

[9] (2017). Annual report 2017. Ministry of Health Malaysia.

[10] (2018). Annual report 2018. Ministry of Health Malaysia.

[11] (2019). Annual report 2019. Ministry of Health Malaysia.

[12] Annual report 2020. Ministry of Health Malaysia.

[13] Annual report 2021. Ministry of Health Malaysia.

[14] Zulaikhah ST, Khalimurrosyid A, Jalu M, Jalu M, Maulana F (2020). Risk factors of leptospirosis in Semarang, Central Java Indonesia: a case control study. Int Med J.

[15] Ab Rahman MH, Hairon SM, Hamat RA, Jamaluddin TZ, Shafei MN, Idris N, Osman M, Sukeri S, Wahab ZA, Mohammad WM, Idris Z, Daud A (2018). Leptospirosis health intervention module effect on knowledge, attitude, belief, and practice among wet market workers in northeastern Malaysia: an intervention study. Int J Environ Res Public Health.

[16] Ridzuan JM, Aziah BD, Zahiruddin WM (2016). Study on seroprevalence and leptospiral antibody distribution among high-risk planters in Malaysia. Osong Public Health Res Perspect.

[17] Hand phone users survey 2021 (HPUS). Malaysian Communications and Multimedia Commission.

[18] (2017). Hand phone users survey 2017. Malaysian Communications and Multimedia Commission.

[19] Ghazaei C (2018). Pathogenic leptospira: Advances in understanding the molecular pathogenesis and virulence. Open Vet J.

[20] Abdullah NM, Mohammad WM, Shafei MN, Sukeri S, Idris Z, Arifin WN, Nozmi N, Saudi SN, Samsudin S, Zainudin A, Hamat RA, Ibrahim R, Masri SN, Saliluddin SM, Daud A, Osman M, Jamaluddin TZ (2019). Leptospirosis and its prevention: knowledge, attitude and practice of urban community in Selangor, Malaysia. BMC Public Health.

[21] Larson RW, Rusk N, Lerner RM, Lerner JV, Benson JB (2011). Intrinsic motivation and positive development. Advances in Child Development and Behavior.

[22] Nozmi N, Samsudin S, Sukeri S, Shafei MN, Wan Mohd WM, Idris Z, Arifin WN, Idris N, Saudi SN, Abdullah NM, Abdul Wahab Z, Tengku Jamaluddin TZ, Abd Rahman H, Masri SN, Daud A, Osman M, Awang Hamat R (2018). Low levels of knowledge, attitudes and preventive practices on leptospirosis among a rural community in Hulu Langat district, Selangor, Malaysia. Int J Environ Res Public Health.

[23] Samsudin S, Saudi SN, Masri NS, Ithnin NR, Hamat RA, Wan Mohd ZW, Nazri MS, Surianti S, Daud AB, Abdullah MN, Noramira N, Osman M (2020). Awareness, knowledge, attitude and preventive practice of leptospirosis among healthy Malaysian and non-Malaysian wet market workers in selected urban areas in Selangor, Malaysia. Int J Environ Res Public Health.

[24] Malaysia health systems research (vol 1). Ministry of Health Malaysia.

[25] (2015). World health statistics 2015. World Health Organization.

[26] Mohamad Marzuki MF, Yaacob NA, Yaacob NM (2018). Translation, cross-cultural adaptation, and validation of the Malay version of the system usability scale questionnaire for the assessment of mobile apps. JMIR Hum Factors.

[27] Zahiruddin WM, Arifin WN, Mohd-Nazri S, Sukeri S, Zawaha I, Bakar RA, Hamat RA, Malina O, Jamaludin TZ, Pathman A, Mas-Harithulfadhli-Agus AR, Norazlin I, Suhailah BS, Saudi SN, Abdullah NM, Nozmi N, Zainuddin AW, Aziah D (2018). Development and validation of a new knowledge, attitude, belief and practice questionnaire on leptospirosis in Malaysia. BMC Public Health.

[28] Rashid NS, Chen XW, Mohamad Marzuki MF, Takshe AA, Okasha A, Maarof F, Yunus RM (2022). Development and usability assessment of a mobile app (Demensia KITA) to support dementia caregivers in Malaysia: a study protocol. Int J Environ Res Public Health.

[29] Gaining consensus among stakeholders through the Nominal Group Technique. Centers for Disease Control and Prevention.

[30] McMillan SS, King M, Tully MP (2016). How to use the nominal group and Delphi techniques. Int J Clin Pharm.

[31] Rosenstock IM (1974). Historical origins of the health belief model. Health Educ Monogr.

[32] Sauro J (2011). Measuring usability with the System Usability Scale (SUS). Measuring U.

[33] Myhealth. Ministry of Health Malaysia.

[34] Anh NQ, Hung LX, Thuy HN, Tuy TQ, Caruana SR, Biggs B, Morrow M (2005). KAP surveys and malaria control in Vietnam: findings and cautions about community research. Southeast Asian J Trop Med Public Health.

